# Epigenetic signature of N-terminal acetyltransferases: a probable mediator of immune and neuropathogenesis in HIV infection

**DOI:** 10.1186/s13041-022-00946-3

**Published:** 2022-08-08

**Authors:** Vaishnavi Sundar, Jay P. McLaughlin, Thangavel Samikkannu

**Affiliations:** 1 Department of Pharmaceutical Sciences, Irma Lerma Rangel College of Pharmacy, Texas A&M University, 1010 W Avenue B, Kingsville, 78363 TX USA; 2 Department of Pharmacodynamics, College of Pharmacy, University of Florida, Gainesville, 32611 FL USA

**Keywords:** Epigenetics, Nt-acetylation, Nt acetyltransferase, HIV, Neurodegeneration

## Abstract

HIV is a major global public threat burdening society, yet the exact mechanism of HIV pathogenesis needs to be elucidated. In the era of epigenetic therapy, N-terminal acetylation (Nt-acetylation) changes induced by viral infection might play a critical role in virus–host interactions in HIV infection. The mitochondrial epigenetic mechanism, predominantly Nt acetylation, holds HIV immunopathogenesis and is vastly unexplored. The challenge is to single out the specific pathological role of NAT changes in HIV-associated neurodegeneration. Therefore, this nano review aims to shine light on Nt acetylation in HIV pathogenesis, which we believe can lead to effective future therapeutic strategies against HIV-associated neurodegeneration.

Human immunodeficiency virus (HIV) remains a major global public health threat, so further understanding the exact mechanisms of pathogenesis during infection would facilitate successful control and treatment. The ability of HIV to infect humans utilizes its ability to hijack immune cells, such as CD4 + T cells, to compromise the immune response. Nevertheless, successful infection eventually progresses to cells of the peripheral and central nervous system (CNS). HIV infection often results in varying degrees of neurocognitive impairment and behavioral deficits collectively called HIV-1-associated neurocognitive disorders (HANDs). Despite effective antiretroviral therapy, HAND is observed in more than 50% of HIV-1-positive individuals [1]. Prominent features of HAND include dendritic damage, reduced spine density, and neuronal loss, which collectively contribute to impaired cognitive function. Significantly, HIV-induced alterations in brain homeostasis and neuronal activity are affected by rapid and pronounced changes in microglial morphology and function, contributing to a neuroinflammatory state. Microglial cells provide an essential immune response in the CNS concomitant with inflammatory brain disease and play a significant role in host defense against invading viruses. The compromise of normal microglial function upon HIV-1 infection is thus an important yet poorly understood contributor to the progression of HAND pathogenesis.

Epigenetic modifications in microglia regulate gene expression and physiological function and include posttranscriptional modifications, such as methylation, phosphorylation, ubiquitination, N-terminal (Nt) acetylation, and lysine acetylation. Among these, Nt acetylation is one of the major posttranslational modifications, catalyzed by N-terminal acetyltransferases or arylamine N-acetyltransferases (NATs). Whereas phosphorylation, methylation, ubiquitination and lysine acetylation regulate the activity of target proteins after translation, modification by Nt acetylation occurs during the translation of the target proteins [2]. Unlike lysine acetylation, Nt-acetylation is an irreversible process that mainly takes place on the ribosome during protein synthesis. NATs preferentially acetylate the N-terminal amino acids of approximately 80% of cellular proteins, thereby accounting for a major source of posttranslational protein modification [3]. NATs are cytosolic enzymes that transfer an acetyl group from acetyl coenzyme A to a drug acceptor substrate. The catalytic triad comprising cysteine, histidine and aspartate in the NAT enzyme contributes to the multifunctional ability of NATs [4]. Nt-acetylation mediates the N-degron (proteolytic pathways that target N-termini) signal, promoting protein degradation, preventing endoplasmic reticulum (ER) translocation, mediating protein complex formation, and linking the metabolic cell state to cell death through acetyl coenzyme A activity (Fig. 1). The essentiality of NATs in humans is stressed by the discovery of a human hereditary lethal disease caused by a mutation in the NAT genes. It was found that Nt-acetylation is crucial for cytosolic retention, as some of the selected Nt-acetylated proteins are not translocated to the ER posttranslationally [6]. Interestingly, NATs can also regulate cellular physiological mechanisms independent of their catalytic activity. For instance, it has been reported that hNaa10p (a NatA subunit) prevents cell migration by regulating DNA methyltransferase 1 activity on E-cadherin, promoting cell proliferation [4].

While minimally studied, Nt-acetylation changes induced by viral infections might play a critical role in regulating the virus − host interaction. The recent demonstration that mice exposed to HIV-1 Tat protein display changes in mitochondrial DNA methylation in isolated brain tissue suggests the feasibility of additional mitochondrial epigenetic modifications occurring in HIV infection [5]. However, the role of whether and how mitochondrial epigenetic status preferentially regulates NATs and the pro-viral immunopathogenesis or anti-viral immunity during HIV infection remains unexplored and a topic of interest (Fig. 1).

The expression of NAT proteins and their specific functions vary depending on the physiological demands of the host cells. For instance, NAT C Nt-acetylates a large variety of proteins and is essential for mitochondrial integrity and function. Interestingly, knockdown of Naa30 (a subunit of the NAT C complex) induces the loss of mitochondrial membrane potential, leading to fragmentation of mitochondria and disorientation of the Golgi apparatus without impacting the ER or functionality of any other organelle [6]. This finding indicates that NAT C is necessary and sufficient to maintain the integrity of the mitochondria and Golgi apparatus. Furthermore, HIV itself contributes to mitochondrial dysfunction, with consequential premature synthesis of free radical superoxide shown to contribute to the progression of neuroAIDS [7, 8]. Moreover, the energy for SWItch/sucrose nonfermentable (SWI/SNF)-mediated epigenetic remodeling relies on the DNA-dependent ATPase activity occurring in the mitochondria, further implicating the pathological importance of mitochondria as mediators of HIV infection. Adding to these pathological events of HIV infection, the energy deficit-associated metabolic dysfunction induced by the virus negatively impacts the energy sensor AMP-activated protein kinases (AMPKs), mitochondrial biogenesis and the activity of the chromatin remodeling complex SWI/SNF, exacerbating the progression of neuroAIDS [9].

Naturally, there are some chromatin complexes that are resistant to HIV-1 integration, and to overcome this barrier, HIV-1 requires nucleosome rearrangement to integrate itself into the host genome facilitated by SWI/SNF chromatin remodelers. Concurrent evidence also suggests that the SWI/SNF protein complex in association with the Forkhead family of transcription factors (FOXO) protein family promotes neurodegeneration [10]. Therefore, exploring the role of mitochondrial Nt acetylation in modulating the ATP-dependent SWI/SNF complex in HIV immunopathogenesis might be expected to identify a novel set of therapeutic targets to treat HAND. Moreover, although the key roles of NATs in particular pathways are being discovered, it remains unclear whether NATs have developed any specific cellular tasks. Prolonged harmful mutations in mtDNA may also result in epigenetic modifications that affect genomic instability, indicating the importance of mitocheckpoint-mediated epigenetic alterations. Since mtDNA is packed with nucleoid proteins and as the mitochondrial genome is devoid of histones, any type of posttranslational modification of histone proteins would not be expected to affect mitochondrial function. On the other hand, mtDNA methylation may play an important role in regulating mitochondrial function, either independently or via a complex mechanism involving nuclear-mitochondrial crosstalk. We have previously shown that HIV-induced changes in mtDNA methylation correspond with mitochondrial genome dysfunction and promote neuroAIDS sequelae. However, the mechanisms regulating Nt-acetylation of mtDNA during HIV infection remain widely unexplored. The major scientific challenges that remain to be deduced are the specific mechanisms that mediate pathological effects arising from NAT alterations in different cellular organelles and assessing their contribution to the progression and continuation of HAND.

**Fig. 1:**
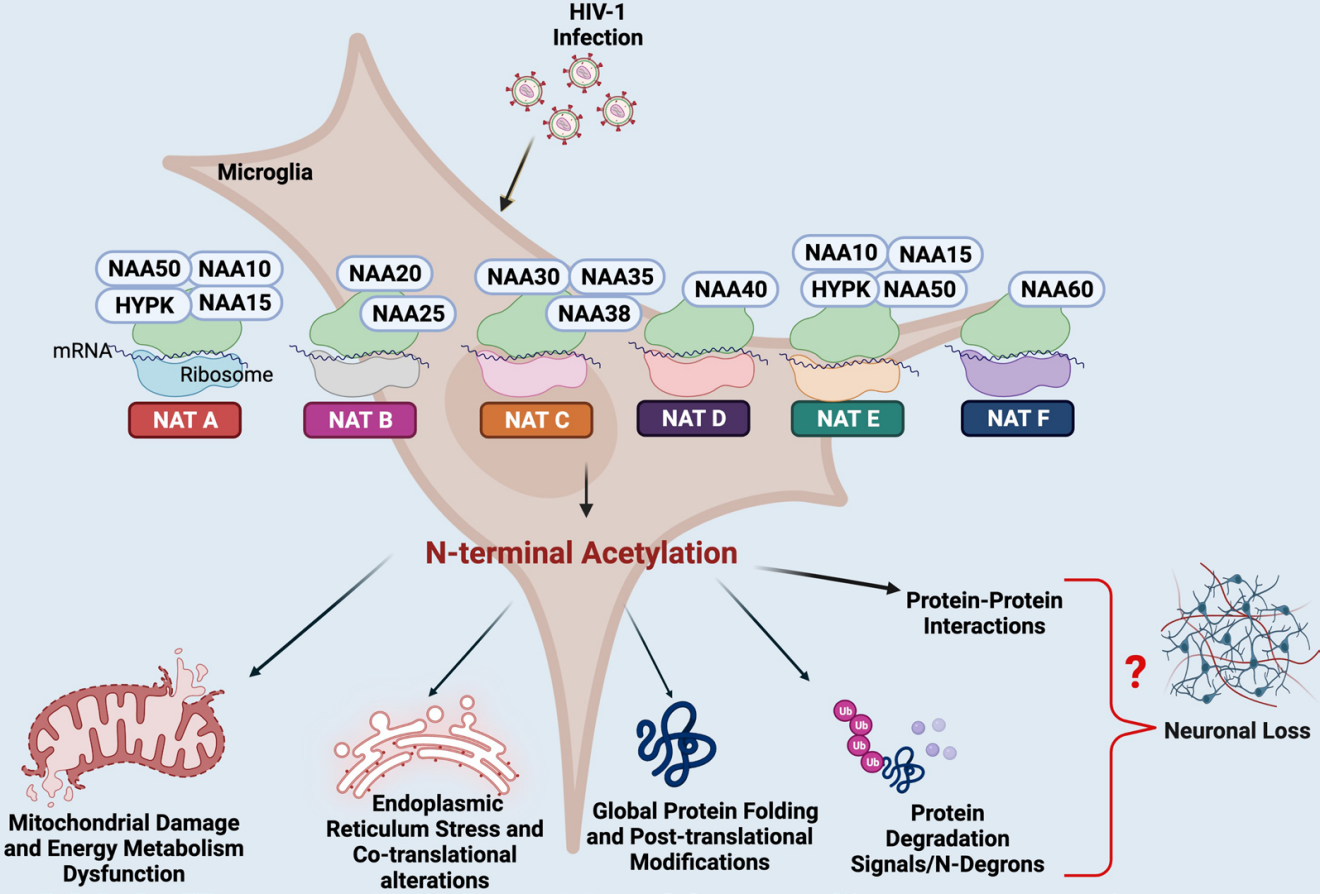
Proposed mechanisms of HIV disease progression influenced by the epigenetic signatures of N-terminal acetylation-impaired immunity and neuropathogenesis in microglial cells. *HIV *human immunodeficiency virus, *NAT* N-terminal acetylation, *NAA* *N*-alpha acetyltransferases, *HYPK* huntingtin interacting protein K

## Data Availability

Data sharing not applicable to this article as no datasets were generated or analyzed during the current study.

## Abbreviations

AIDS: Acquired immunodeficiency syndrome
AMPK: AMP-activated protein kinase
ATP: Adenosine tri phosphate
CNS: Central nervous system
ER: Endoplasmic reticulum
HAND: HIV-1 associated neurocognitive disorders
HIV: Human immunodeficiency virus
FOXO: Forkhead family of transcription factors
NAT: N-Terminal acetyltransferases
NAA: N-alpha acetyltransferases
HYPK: Huntingtin interacting protein K
SWI/SNF: SWItch/sucrose nonfermentable
